# Prevalence of intestinal parasitosis and associated risk factors among school children of Saptari district, Nepal: a cross-sectional study

**DOI:** 10.1186/s41182-020-00261-4

**Published:** 2020-08-24

**Authors:** Ranjit Gupta, Binod Rayamajhee, Samendra P. Sherchan, Ganesh Rai, Reena Kiran Mukhiya, Binod Khanal, Shiba Kumar Rai

**Affiliations:** 1 ShiGan International College of Science and Technology, Kathmandu, Nepal; 2 Department of Infectious Disease and Immunology, Kathmandu Research Institute for Biological Sciences (KRIBS), Lalitpur, Nepal; 3 School of Optometry and Vision Science, Faculty of Science, UNSW, Sydney, NSW 2052 Australia; 4 Department of Environmental Health Sciences, School of Public Health and Tropical Medicine, Tulane University, New Orleans, LA USA; 5 National Institute of Tropical Medicine and Public Health Research, Kathmandu, Nepal

**Keywords:** Parasitosis, Risk factors, *Giardia lamblia*, School children, *Terai* area, Nepal

## Abstract

**Background:**

Intestinal parasitosis, caused by both helminths and protozoans, are among the most prevalent infections, especially in developing countries. Enteric parasites continue to be a major cause of parasitic diseases which is the most common among street and school going children with poor sanitation. This cross-sectional study was carried out to determine the prevalence and potential risk factors of intestinal parasitosis among school going children of two schools of Saptari district of southern Nepal. Stool samples were collected in a clean, dry, screw-capped, and wide-mouthed plastic container, fixed with 10% formal-saline solution, and transported to the laboratory for further microscopic analysis by following concentration technique.

**Results:**

Out of the 285 stool samples analysed, 94 (33%) were positive for the parasitosis. Presence of intestinal parasites was marginally more in rural school (44.6%) than in urban (30%) (*P* < 0.05). *Giardia lamblia* was highly prevalent (15.4%) followed by *Entamoeba histolytica*-like (7.7%), *E*. *coli* (7%), *Ascaris lumbricoides* (1.8%), and *Hymenolepis nana* (1.08%), respectively. Children of the age group 11–15 years were highly affected (44.2%) compared to younger age groups. The findings of intestinal parasitosis in the study population were statistically significant with family income, hand-washing habit, type of drinking water, and availability of a toilet facility at home (*P* < 0.05). Over 85% of infection was associated with parasitosis that indicated mainly waterborne infection rather than soil-borne helminths.

**Conclusions:**

Poor hygiene measures and farming occupation are identified as major risk factors of parasitic infections, so sanitation especially focusing on safe drinking water along with multi intervention strategies must be emphasized in the Saptari district of Nepal to reduce the burden of parasitic diseases in school children.

## Introduction

Intestinal parasitic infections (IPIs) associated with protozoa and helminths have been a common public health problem, particularly in developing nations like Nepal [1], where children are more commonly infected resulting in both physical and mental retardation after the infection [2]. As per World Health Organization, more than 270 million pre-school children and over 600 million school going children are living in the area where parasitic diseases are more prevalent and there is an urgent need of disease control interventions [3]. More than 880 million children need treatment for parasitic infections, and notably, school going children, farmers, and rural villagers are at a high risk of having intestinal parasitosis due to the unhygienic conditions [4].

Infectious diseases were the leading cause of morbidity and mortality in Nepal and have been listed as the ‘top ten diseases’ of the country until the year 2000, but have shown a declining trend, especially vaccine-preventable diseases in the last 10–15 years of period [5]. Intestinal parasites are the leading cause of diarrhoea which is transmitted faeco-orally when we consume contaminated food and water. Most of the diarrhoeal infections result in malnutrition, abnormal physical growth, and anaemia [6]. In Nepal, intestinal parasitosis has been a major public health issue for a long time [7], and the prevalence varies from 13 to 81% [8, 9] while the rate is even a hundred percent in some rural areas [10]. However, the prevalence rate has decreased in recent years [7]. Nepal began the periodic deworming programme in the fiscal year 2006/2007 [11] which has been combined with national vitamin A supplementation targeting school going children. Unfortunately, nationwide data on the effectiveness of the campaign and its coverage is insufficient [12]. The prevalence of intestinal parasitosis among school going children, however, has reduced significantly (61% in the late 1990s to around 20% during recent years) [13] and appears to be due to both deworming and improvement of sanitary as well as hygienic practice during this period. Also, this has been attributed to the countrywide ‘open defecation free movement’ launched in 2010 by the government of Nepal [9]. In this context, this cross-sectional study was conducted to determine the prevalence of intestinal parasitosis among school children of two public schools from the Saptari District of Rajbiraj Municipality (urban area) and Mahadeva Village Development Committee (rural area), which is in the southern part of Nepal (*Terai*/plain area). Hence, the present study aimed to determine the prevalence and risk factors associated with intestinal parasitosis among school children of *Terai* region which is considered poor in terms of sanitation and personal hygiene.

## Materials and methods

### Study type and area

**Fig. 1:**
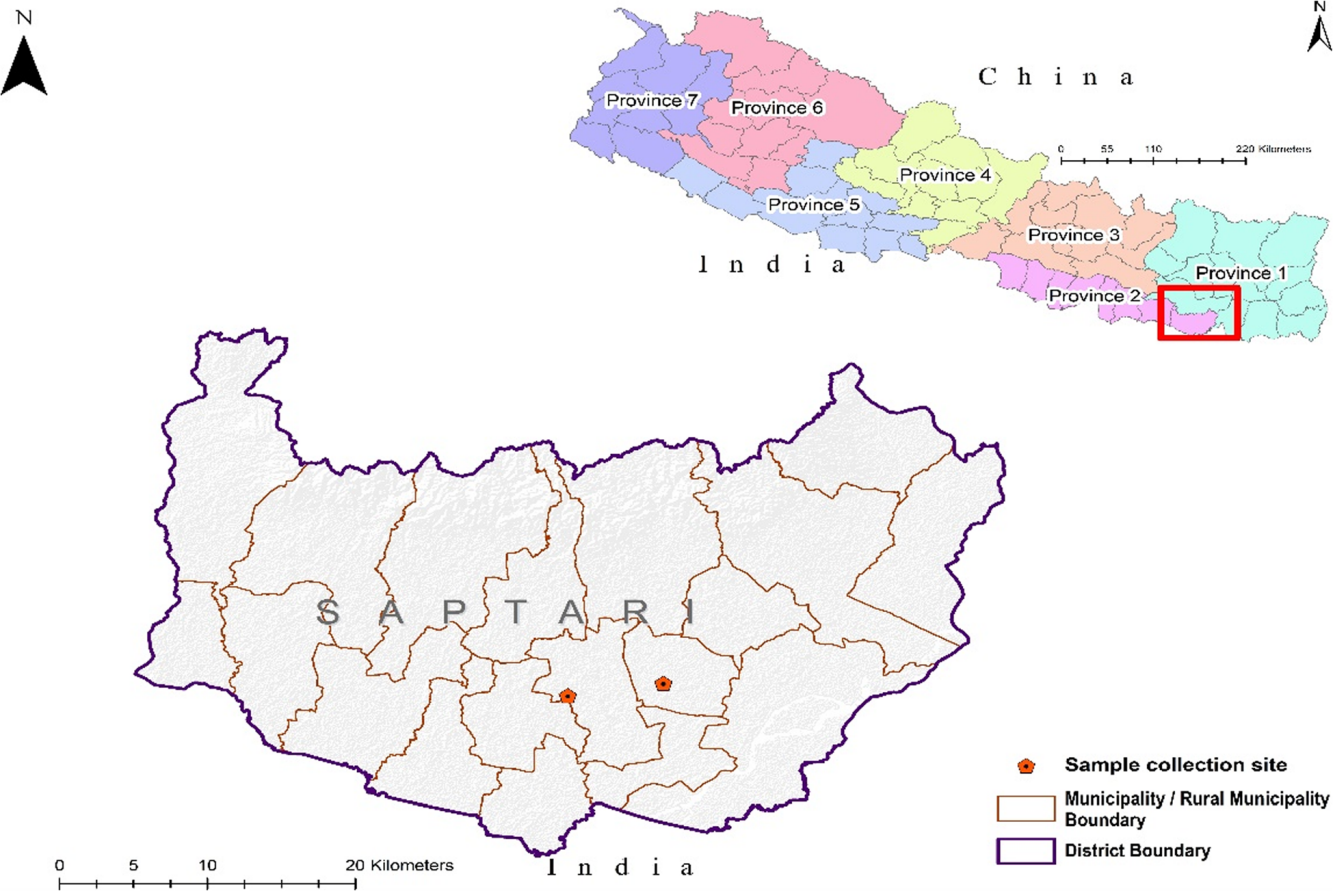
GIS map showing the geographic location of Saptari district, Nepal

Two school-based cross-sectional studies were carried out between May and October 2017, where children of age up to 15 years from diverse socioeconomic status and ethnic groups were included. More cases of diarrhoeal diseases are reported during the rainy season which falls between June and August in Nepal, so we have chosen this season to collect the study samples. The study site was the Saptari district of province 2, Nepal, one of the densely populated districts, which is about 300 km to the south from the capital city Kathmandu and is also bordered with India (Fig. 1).

### Sample collection and observation

**Fig. 2:**
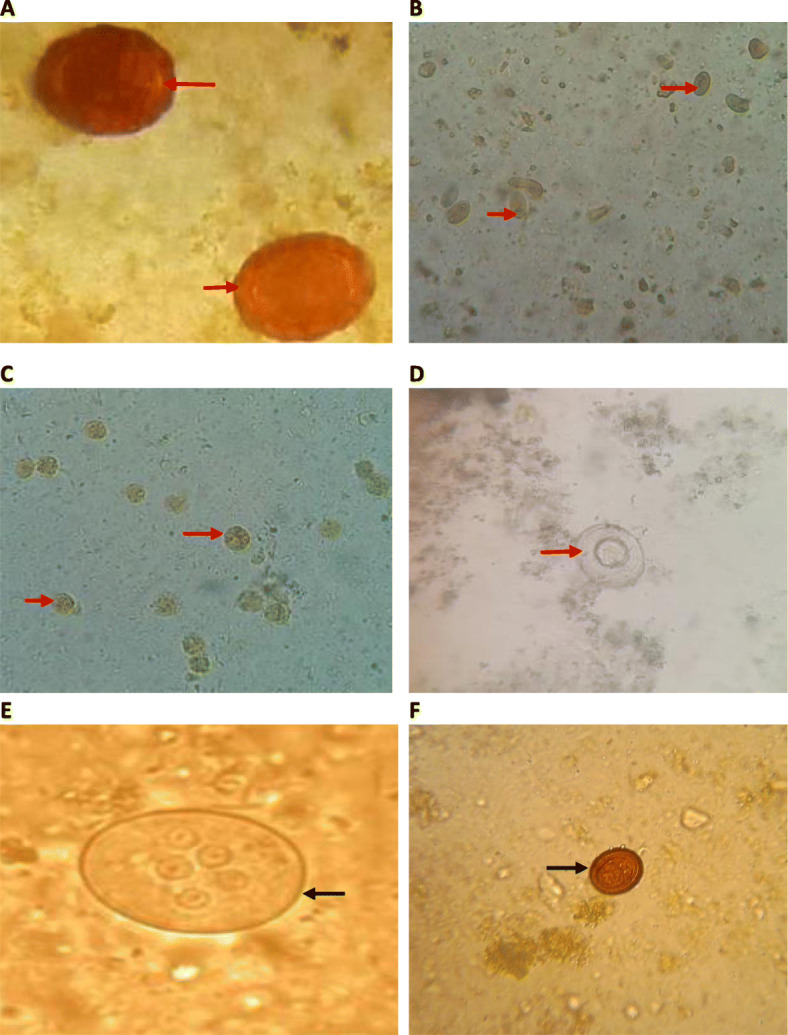
Microscopic observation of intestinal parasites. **a** Eggs of *A*. *lumbricoides* (sample code-1126S). **b** Oval shape cysts of *G*. *lamblia* (sample code-1033S). **c** Cysts of *Entamoeba histolytica*-like protozoa (sample code-1013S). **d** Egg of *Hymenolepis nana* (sample code-1056S). **e** Cyst of *Entamoeba coli* (sample code: 1106S). **f** Egg of *Taenia* spp. (sample code-1217S). Solid arrow indicates egg or cyst of parasite

Stool samples were collected from school going children aged up to 15 years in two locations: Durga Secondary School in the urban area and Mahadeva Primary School in the rural area of Saptari district in southern Nepal (Fig. 1). The sample was collected at the same time from two schools. Enrolled students were categorized into 3 age groups: up to 5, 6–10, and 11–15 years. A total of 285 children were enrolled in this study without any visible physical disabilities. Informed consent was taken from the school principal and science/health teachers at respective schools, before stool sample collection. Due to student numbers being fewer in the rural area compared to the urban area, only 92 children were enrolled from the rural school. A questionnaire on age, gender, sanitary condition (availability of toilets at home), source of drinking water, hygienic practice of children (hand-washing habits), family occupations, and medical history (vomiting, nausea, and abdominal pain) was filled by research personnel at the time of sample collection. Laboratory findings were recorded and stratified against the location, age, gender, and other demographic information of the study population. Instruction for stool collection was provided in a local language then well-labelled stool containers and application sticks were given to each student. The next day, each sample was thoroughly checked for its quantity (2 gm) and labelling. Stool samples were collected in clean, dry, screw-capped, and leak-proof plastic containers fixed in an equal volume of 10% formal saline solution. Collected stool samples were then immediately transported to ShiGan International College in Kathmandu for the laboratory examination. Stool samples were examined by the concentration method employing formal-ether sedimentation followed by microscopic examination for cysts and oocysts of protozoa and eggs of helminth parasites (Fig. 2). A cotton gauze was used to filter the formalin-fixed stool sample (3-4 ml) in the test tube, and then 3-4 ml of diethyl ether was mixed and shaken for about 4–5 min. Then, it was centrifuged for 15 min at 3000 rpm, and iodine solution was used to mount the sediment. Microscopic examination was done for the observation of cyst, trophozoites, and ova of parasites present in the collected stool specimens using × 10 and × 40 magnification. The presence of any blood, mucus, colour, and consistency was examined macroscopically [14, 15]. Examination (macroscopic and microscopic) of all specimens was performed following the standard operating procedures (SOPs) as recommended by the WHO [16].

### Quality control

All laboratory instruments like microscope, centrifuge, staining reagents, sample collection containers, and transporting systems were checked regularly to ensure the correct functioning of each material for the consistency of results as an internal quality control. Each sample was double-checked for correct labelling and quantity. To avoid the examiner bias, each specimen was observed independently by two microbiologists of ShiGan International College, Kathmandu.

### Statistical analysis

Data analysis was done by the chi-square test using SPSS-16 version (IBM SPSS Statistics). The chi-square test was used to evaluate apparent differences for significance at 95% confidence level. Results were considered significant if the *P* value was less than 0.05. The presence of parasites in stool specimens with respect to individual habits and demography were evaluated by using chi-square values. During data collection, any missing information in questionnaire sheets was regularly checked.

## Results

### Incidence of parasitosis

A total of 285 school children of age up to 15 years from two different schools were included in this study as the study population consisted of 172 (60%) male and 113 (40%) female children (the ratio of male to female was 1:1.5). Among 285 stool samples analysed, the total prevalence of parasitosis among the study population was 33% (94/285) where 29.7% were male and 38.1% were female children (*P* = 0.14). There were 7 positive cases (7/94, 7.4%) from the same family. Out of 94 positive samples, 91.5% (86/94) were protozoa, and the remaining 8.5% (8/94) were helminths parasites. The overall prevalence of protozoal and helminthic cases was 30.2% and 2.8%, respectively. Six species of intestinal parasites were reported: *Giardia lamblia*, *Entamoeba histolytica*-like, and *Entamoeba coli* as protozoan species and *Ascaris lumbricoides*, *Hymenolepis nana*, and *Taenia* spp. as helminthic species (Table 1, Fig. 2a–f). *Entamoeba histolytica* cannot differentiate from *E*. *dispar* or *E*. *moshkovskii* by microscopic examination because of their similarity in morphology, so *E*. *histolytica*-like is used in this study. *G*. *lamblia* (46.8%) was the most predominant species followed by *Entamoeba histolytica*-like (23.4%), and *Entamoeba coli* (21.3%) where the overall prevalence of *G*. *lamblia* was 15.4% and *A*. *lumbricoides* was the most predominant (5.3%) species among helminths isolates. Among three different age groups of children, the highest rate of parasitosis was seen in the 11–15 years (44.2%) followed by 5–10 years (31.6%) and up to 5 years (23.2%), respectively, and more cases were reported from the school of rural area (44.6%, Mahadeva school) than the urban area (30%, Durga school) (Supplementary file, Fig. 1, Tables 1 and 2) and the result was statistically significant (*P* = 0.005484). None of the processed samples showed mixed infection of parasites.

**Table 1:** Age-wise distribution of intestinal parasitic infections

**Intestinal parasites**	**Up to 5 years (*n*** **= 95)**	**6–10 years (*n*** **= 95)**	**11–15 years (*n*** **= 95)**	**Total [*n*** **(%)]**
**Protozoa**	**19**	**28**	**39**	**86 (30.2)**
*Giardia lamblia*	11	13	20	44 (15.4)
*Entamoeba histolytica*-like	3	7	12	22 (7.7)
*E*. *coli*	5	8	7	20 (7.0)
**Helminths**	**3**	**2**	**3**	**8 (2.8)**
*Ascaris lumbricoides*	1	1	3	5 (1.8)
*Hymenolepis nana*	1	1	0	2 (0.7)
*Taenia* spp.	1	0	0	1 (0.4)
**Total [*n*** **(%)]**	**22 (23.2)**	**30 (31.6)**	**42 (44.2)**	**94 (33)**

**Table 2:** School-wise distribution of intestinal parasitic infections

**Intestinal parasites**	**School of rural area [*n*** **(%)]** **92 (32.3)**	**School of urban area [*n*** **(%)]** **193 (67.7)**
**Protozoa**	**39 (92.9)**	**47 (90.4)**
*Giardia lamblia*	16 (38.1)	28 (53.9)
*Entamoeba histolytica*-like	10 (23.8)	12 (23.1)
*Entamoeba coli*	13 (30.9)	7 (13.4)
**Helminths**	**3 (7.1)**	**5 (9.6)**
*Ascaris lumbricoides*	2 (4.8)	3 (5.8)
*Hymenolepis nana*	1 (2.4)	1 (1.9)
*Taenia* spp.	0	1 (1.9)
**Total [*n*** **(%)]**	**42 (45.7)**	**52 (26.9)**

### Association of parasitosis with risk factors

In this study, parasitic infection in school going children was dependent variable while socio-demographic and personal behavioural features were independent variables. Children with hand-washing habits in their school and home were less likely to have intestinal parasitic infection (27.2%) as compared to those with no hand-washing habits in their school and home (67.2%), and the result was statistically significant (*P* = 0.00001). Children who used groundwater (tube well/boring water) for drinking purposes have more cases of intestinal parasites (68/94) than children who used tap water for drinking (26/94), but there was no statistically significant difference (*P* = 0.209). Children without toilet facility at home were most likely (42.9%) to be infected than those with the toilet facility (29.8%), and the result was statistically significant (*P* = 0.043052). The prevalence of parasitosis was more among children who had gastrointestinal pain than without pain and other symptoms (*P* < 0.05). The higher infection was found in those children whose family profession was farming compared to other occupations (*P* < 0.05) (Table 3).

**Table 3:** Potential risk factors associated with the prevalence of parasites among study population

**S.N.**	**Risk factors**	**Collected samples (*n*** **= 285), %**	**Positive numbers (*n*** **= 94), %**	**Chi-square (*χ***^**2**^**) value**	***P*** **value**
**1.**	**Gender**				
	Male	172 (60)	51 (29.7)	2.17	0.14
	Female	113 (40)	43 (38.1)		
**2.**	**Study site**				
	Urban	193 (67.7)	54 (30.0)	7.71	0.005484
	Rural	92 (32.3)	41 (44.6)		
**3.**	Toilet facility (at home)*				
	Yes	215 (75.4)	64 (29.8)	4.09	0.043052
	No	70 (24.6)	30 (42.9)		
**4.**	**Drinking water source (school and home)**				
	Tube well/boring water	192 (67.4)	68 (35.4)	1.57	0.209
	Tap water	93 (32.6)	26 (28.0)		
**5.**	**Hand-washing habit (school and home)**				
	Yes	169 (59.3)	46 (27.2)	44.82	0.00001
	No	116 (40.7)	78 (67.2)		
**6.**	**Family occupation**				
	Business	47 (16.5)	13 (27.7)	8.65	0.034251
	Farming	107 (37.5)	46 (43.0)		
	Office	58 (20.4)	18 (31.0)		
	Others	73 (25.6)	17 (23.3)		
**7.**	**Gastrointestinal pain/symptoms**				
	Yes	196 (68.8)	76 (38.8)	9.52	0.002022
	No	89 (31.2)	18 (20.2)		

*There was a toilet facility in both schools

## Discussion

The spread of intestinal parasites solely depends on the status of sanitation and the socio-economic setting in the community. The prevalence of intestinal parasitic infections is determined by multiple factors of our living society like occupation, hygiene condition, economic status, the facility of toilets at home, and drinking water, among others. This study attempted to determine some potential risk factors associated with the prevalence of parasitosis among school going children of two schools based in Saptari district. In this study, one third of enrolled school children (33%, 94/285) was found to be infected with intestinal parasites and a similar rate of prevalence was reported by Shah et al. [17] while some other studies have reported a low rate of intestinal parasites in school going children from different parts of the country [6, 18, 19]. Higher prevalence of IPIs in the study might be due to the contamination of the drinking water supply by the parasites and poor sanitation practice in the study sites [20]. On the other hand, Shreshtha et al. have reported 39.7% of IPIs among school children of two schools from central Nepal [18]. Similarly, very high rates of IPIs were reported in school children from different countries [21–24]. Another study from Morang, a neighbour district of Saptari, reported 83.3% of stool samples with helminth parasites in school children where *A*. *lumbricoides* (50.92%) was the most detected parasite followed by *Ancylostoma duodenale* (44.56%) and *Trichuris trichiura*, respectively [25]. The prevalence of IPIs was 13.9% among school children in another study conducted in Parsa, a district of *Terai*, where more girls were infected (19%) than boys (10%). The most common parasite was *E*. *histolytica* (36.0%) followed by *A*. *lumbricoides* (28.0%), and children of illiterate and farmer parents were at higher risk of infection [26].

In this study, the incidence of protozoan parasites and helminth parasites were 30.2% (86/94) and 2.8% (8/94), respectively. Previous studies have also reported more cases of protozoa than helminthic parasites [1, 6]. In our study, *G*. *lamblia* was positive in 44 out of 94 cases (Fig. 2a). Globally, infection of *G*. *lamblia* is highly associated with the low level of sanitation and the most common cause of diarrhoeal illness which is normally called giardiasis. In essence, *G*. *lamblia* is found in contaminated foods, water, and soil [27] which is mainly transmitted to a healthy individual via contaminated foods and water. *Giardia* cysts were reported up to 43% of drinking water samples examined in Kathmandu Valley, which suggests contaminated water as the main vehicle of diarrhoeal infection in Nepal [7]. The second highest case behind *G*. *lamblia* was *E*. *histolytica*-like parasite which has been reported from different places of the country [6, 28]. *A*. *lumbricoides* was the most reported helminth in this study, and similar findings were reported by Shrestha et al. and Khadka et al. [8, 29]. On the other hand, Shreshtha et al. and Tandukar et al. reported that *H*. *nana* was the highly detected helminth among school going children [6, 30]. Our study showed limited cases of helminths compared to the protozoa, in contrast to the other reported studies elsewhere [31, 32]. The nationwide deworming programme along with vitamin A supplement of the Nepal government targeting school-aged children may have played a key role in reducing the helminth parasites in our study because helminthic infections are mainly linked with nutritional insufficiencies especially vitamin A and iron [6]. Additionally, there may be association of lower prevalence of helminths with mass antihelminthic drugs administered in the last 6 months of the sample collection period.

Our findings suggested that there is high level of faecal contamination and proper management of safe drinking water supply is an urgent need. A high prevalence (35.4%) of IPIs was observed among children who used groundwater (tube well/boring) for drinking purposes as compared to tap water. School going children especially in rural areas of Nepal are highly susceptible to IPIs because of poor sanitation practices, which need effective interventions to control intestinal parasitosis in these children [33]. Due to the lack of sufficient data on the prevalence of IPIs and demographic factors which could impact the spread of infection, proper action and plans are not effective enough to reduce the IPIs particularly in resource-limited settings [34]. None of the analysed stool samples showed mixed infection of parasites and helminths. Similarly, less prevalence (6.5%) of mixed parasitic infection was reported by Pradhan et al. (2013) among public school-aged children in a village of Kathmandu district [33]. In a cross-sectional study conducted among 2372 school students in Ethiopia, only 3.4% of children were infected by mixed parasites [35]. The variation in mixed parasitic infections may be due to hygiene conditions of the studied population and differences in concentration of parasites in processed samples [36]. The findings of our study showed girls (38.1%) have the higher infection than boys (29.7%) (*P* > 0.05), and the finding was inconsistent with the results reported by Tandukar et al. (2013) where male children were more infected [6]. Socio-behavioural activities and awareness of good health manners greatly affect the association of IPIs with gender. The infection rate was higher in rural area (44.6%) than in urban areas (30%). This might be due to the relatively poor sanitary condition in the rural area of the study site. Other studies have also reported higher rates of IPIs in rural areas of Nepal [17, 30]. Additionally, low-income families remain in remote areas, so this study suggests there is a direct relationship between the rate of parasitic infections and the socio-economic situation of a family. Some of the studies reported even from the slum sites of Kathmandu Valley have shown a higher rate of IPIs [1], which reflects the poor sanitation and unhygienic behaviour of people living in slum areas.

The age of an individual is considered as a potential risk factor for IPIs. In this study, a high rate of IPIs was found in children of the age group 11–15 (44.2%, 42/94) followed by 6–10 (31.6%, 30/94), and up to 5 years (22, 23.2%), respectively (Supplementary figure 1). Similar findings were reported by Shrestha et al. and Tandukar et al. [6, 28]. Older children get more exposed to outdoors activities and likely to eat fast food from the markets, which could be an important risk factor for the IPIs. In contrast, high prevalence of IPIs was reported in children of the lower age group in schools of Kathmandu Valley [29], and a similar finding have been reported from Ghana [36] and Pakistan [37]. This could be due to more awareness of personal sanitation and hygienic behaviours among older children as compared to children of low age group.

Additionally, the facility of toilets and hand-washing habits are also considered important risk factors for the incidence of parasitic infection. The findings showed less IPIs among children who had toilet facilities at home and regular hand-washing habit as compared to children who had no toilet facility at home and not having regular hand-washing behaviour (*P* < 0.05). Similar findings were also reported from other parts of Nepal and different countries [38–40] where children with no hand washing habits and without toilet facility were at higher risk of intestinal parasites infection. Therefore, appropriate hand-washing habit especially in school aged children, with an adequate frequency, is considered as an essential preventive measure to protect from many infectious diseases including IPIs [41]. In this study, we have considered the hand-washing practice of a child: if he/she had the habit of hand-washing every time (always) with soap/disinfectant and water before eating any food, after touching rubbish, after using the toilet, and after playing both in school and home. During sample collection time, some open defecation places were also found mainly in rural area. This might be the reason for contaminating the drinking water supply and food processing plants of the region while the government of Nepal has initiated ‘open defecation free movement’ in 2010 and has declared many places as open defecation free districts so far [13]. In this study, the majority of the children’s family occupation was farming (37.5%, 107/285). Children whose parents were involved in farming (43%) were found to be a potential risk factor of IPIs followed by office (31%), business (27.7%), and others (25.6%). The occurrence of high prevalence in children whose parents were farmers might be due to frequent behaviours of exposure to the soil and other organic fertilizers [28]. Basically, abdominal pain is a typical symptom of IPIs, so abdominal pain is taken as a marker for clinical diagnosis of parasitosis in children which has also been proved by this study. Abdominal pain was pointedly observed in children with parasitosis (*P* = 0.002022).

Present findings showed one third of the school children was found to be infected with parasites; however, only one type of parasite was implicated in an individual child. Over 85% of infection was associated with protozoa infections that indicated mainly waterborne infection rather than soil-borne helminths. Therefore, sanitation especially focusing on safe drinking water must be emphasized in the Saptari district of Nepal. Due to rapid urbanization in major cities of Nepal and improper sewage management system, faecal contamination of drinking water is significant. Additionally, purification of drinking water is not maintained and regulated properly at the community level. Furthermore, poor hygienic and sanitary practices augment high prevalence of parasitic infections in Nepal although regular deworming and no-open defecation campaign are in the front line. Effective efforts from concerned stakeholders to improve sewage drainage system and to improve toilets facilities that are being used in homes and schools will certainly reduce the burden of parasitic infection. Introduction of a simple, safer, and cost-effective onsite water treatment facility at household or community level along with knowledge on personal hygiene and sanitation can sharply reduce the number of parasitic infections among school going children in Nepal.

## Conclusion

Despite the nationwide deworming programme run by the government of Nepal, we found a high rate of intestinal parasitosis among school going children, which indicates a greater focus on multiple intervention strategies by improving hygienic practices and safe drinking water especially in rural parts of the nation. This study has identified risk factors such as lack of toilet facility at home, poor hygienic behaviour, poverty, and the use of unsafe drinking water which are associated with IPIs in the study population. This study suggests a need for multi-sectoral plans along with awareness of good health practices to children and their family which could narrow down the burden of parasitic infections in school going children.

## Limitations

Due to limitation in funding and research time, we could not enrol a large number of school children from the study area. In addition, this study represents only short duration data so further studies with a longer period should cover a greater number of participants and other associated predisposing factors of IPIs from different parts of the nation which can determine the burden of IPIs in school children of Nepal.

## Supplementary information

**Additional file 1.** Supplementary figure 1. Prevalence of enteroparasites in different age groups of study population.

## Data Availability

All the data obtained and analysed are included in this manuscript.
