# Supplementary material for: Prevalence of intestinal parasitosis and associated risk factors among school children of Saptari district, Nepal: a cross-sectional study
Source: Trop Med Health. 2020 Aug 24;48:73. doi: 10.1186/s41182-020-00261-4 (PMC7444033; doi:10.1186/s41182-020-00261-4)
Supplement: Supplementary file 1 — Additional file 1. Supplementary figure 1. Prevalence of enteroparasites in different age groups of study population. [file 41182_2020_261_MOESM1_ESM.docx]

**Supplementary figure 1:** Prevalence of enteroparasites in different age groups of study population
